# Association of parental depression with adolescent children’s psychological well-being and health behaviors

**DOI:** 10.1186/s12889-024-18337-9

**Published:** 2024-05-27

**Authors:** Sung-In Kim, Sung Min Kim, Sun Jae Park, Jihun Song, Jaewon Lee, Kyae Hyung Kim, Sang Min Park

**Affiliations:** 1 Department of Psychiatry, Seoul National University Bundang Hospital, Seongnam, Republic of Korea; 2 Department of Biomedical Sciences, Seoul National University Graduate School, College of Medicine, 101 Daehak-ro, Jongno-gu, Seoul, Republic of Korea; 3 Department of Psychiatry, University of Rochester Medical Center, Rochester, NY USA; 4 Department of Family Medicine, Seoul National University Hospital, Seoul National University College of Medicine, 101 Daehak-ro, Jongno-gu, Seoul, Republic of Korea; 5 Home Healthcare Clinic, Public Healthcare Center, Seoul National University Hospital, 101 Daehak-ro, Jongno-gu, Seoul, Republic of Korea; 6 Department of Transdisciplinary Medicine, Seoul National University Hospital, Seoul, Korea

**Keywords:** Adolescent, Parental depression, Mental health, Health behaviors

## Abstract

**Background:**

Parental depression is a significant problem that negatively affects parents’ welfare and influences family dynamics, children’s academic and health behaviors, and mental health. However, there is limited evidence regarding the impact of the parental depression into the children’s’ psychological and physical wellbeing on Asian cultures. This study examined the psychological burdens and health behaviors of adolescent children with parents with depression in the Republic of Korea.

**Methods:**

We conducted a cross-sectional study using data from the Korean National Health and Nutrition Examination Survey (KNHANES) spanning 2013 to 2021 to compare health behaviors and mental health outcomes between 203 adolescent children with parents diagnosed with depression and 3,856 control adolescents aged 12–19 years.

**Results:**

Following multivariate adjustments, the risk of depressive mood for more than two weeks was significantly increased in boys with parental depression (adjusted Odds Ratio [aOR] = 2.05, 95% Confidence Interval [CI] = 1.91–3.52) and adolescents with parents with moderate-to-severe depression (aOR = 2.60, 95% CI = 1.17–5.77). Adolescents with parental depression reported significantly worse subjective health status (aOR = 1.88, 95% CI = 1.05–3.36) and higher stress levels (aOR = 1.91, 95% CI = 1.33–2.76). Additionally, when parental depression was present and the time since depression diagnosis was more than five years, adolescents with parental depression exhibited even poorer subjective health status and higher stress levels.

**Conclusions:**

The study found that adolescents whose parents experienced depression had poorer mental health than those whose parents did not have mental health issues. These findings emphasize the importance of providing support for the mental health of adolescents in families affected by parental depression.

**Supplementary Information:**

The online version contains supplementary material available at 10.1186/s12889-024-18337-9.

## Background

Depression is characterized by a persistently-low mood and an impairment of various aspects of one’s ability to function mentally — including thought content and process, sleep, concentration, energy levels, and physical activity [1]. The World Health Organization has reported that depression is gradually increasing worldwide and is expected to become the most burdensome disease across humanity by 2030 [2].

Parental depression is a significant problem that negatively affects parents’ welfare and influences family dynamics, children’s academic and health behaviors, and mental health. Studies have shown that parental depression increases stress in children [3], suicide rates [4], and depressive symptoms [5–7]. The severity and duration of parental depression, as well as its treatment status, may also affect children’s well-being [5, 8–10].

Adolescents are particularly vulnerable to the effects of parental depression [7]. This stage of life involves rapid physical and emotional development, and external factors such as changes in family structure, school stress, and peer relationships can contribute to emotional problems [11]. Therefore, depression in parents (who act as primary caregivers) affects parenting styles, family dynamics and emotional support, all of which can affect adolescent health [8, 11]. The chronicity of parental illness, family structure, income level, and other factors that affect family dynamics can also exacerbate the impact of parental depression on children [3, 12, 13].

Although studies on the physical and psychological well-being of children with parental depression have been conducted in Western countries [3, 5, 14], there is limited evidence regarding their impact on Asian cultures [15]. Specifically, parents in Asian countries exhibit higher parenting stress compared to Western parents [16]. The increasing prevalence of single-parent families in Asian countries, driven by rapid changes in family structure [17] and the burden of education expenses [18], contributes to the gradual rise in parental depression. Considering that East Asia exhibited the highest rates of parental depression compared that Europe showed the lowest rates [19], it becomes essential to explore how parental depression affects adolescnet children in Asian contries with reflecting the cultural context in Asia.

Korean adolescents already have the highest rates of suicide (23.6 per 100,000 population) and depressive mood among compared to other OECD countries [20–22]. In addition, through the COVID-19 pandemic period, physical activity of adolescents has been declining, and BMI is gradually increasing [23] even though there has been a decreasing trend in smoking and alcohol use among Korean adolescents’ health behaviors. These results highlights the importance of understanding the relationship between parental depression and the health and well-being of adolescent children is crucial for developing preventive strategies. Therefore, this study used data from the Korean National Health and Nutrition Examination Survey (KNHANES) to investigate how parental depression affects the health behaviors and mental well-being of adolescent children.

## Methods

### Study design, settings and population

We conducted a cross-sectional study using data from the sixth (2013–2015), seventh (2016–2018), and eighth (2019–2021) waves of KNHANES [24]. The KNHANES is a nationwide survey that examines the nutritional and overall health statuses of the Korean population and provides comprehensive information on sociodemographic characteristics, health behaviors, and overall well-being. The study protocol was approved by the Ethics Committee of Seoul National University Hospital (IRB number E-2304-102-1425).

**Fig. 1:**
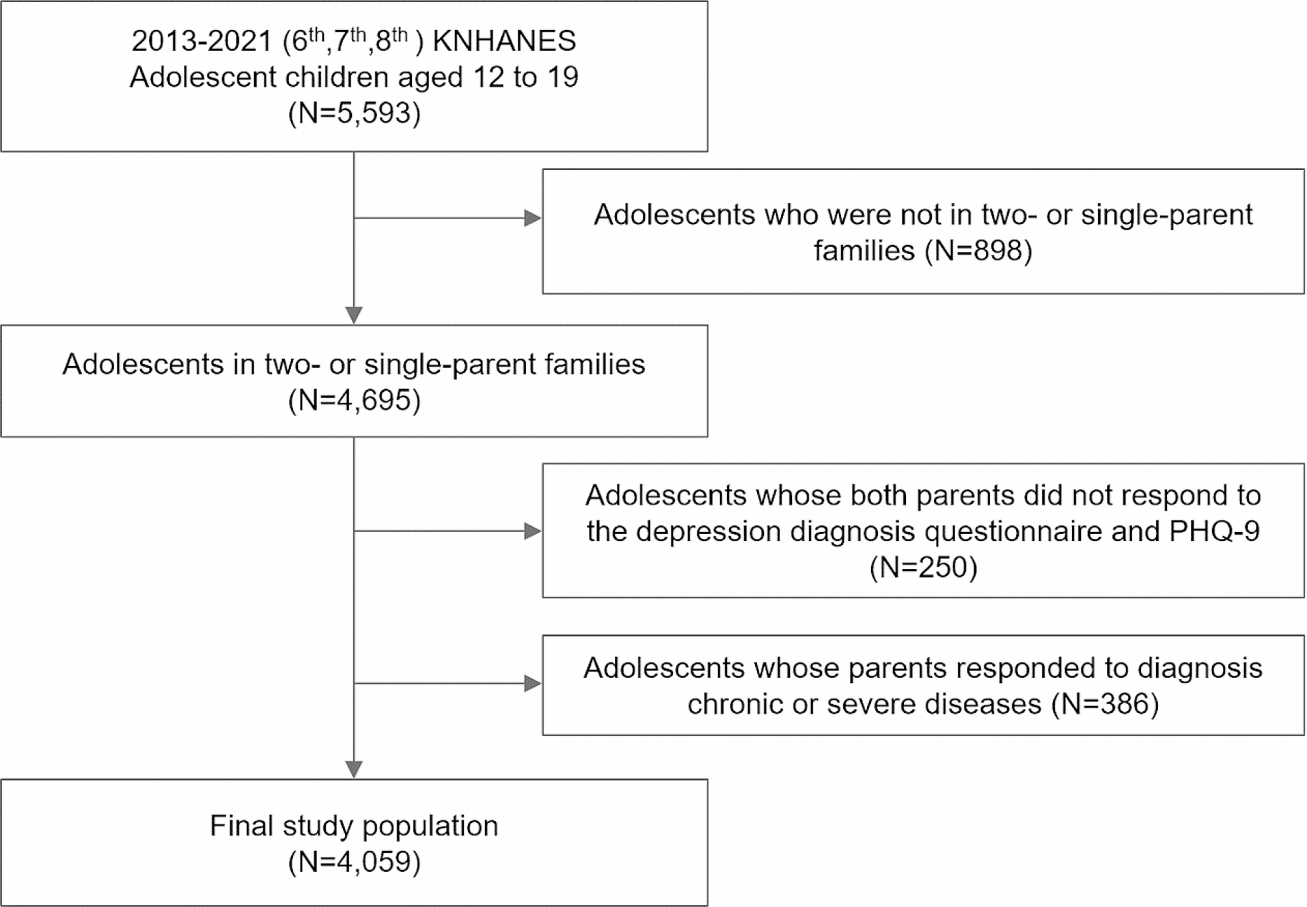
Study population

To account for family structure, the initial sample consisted of 4,695 adolescents (aged 12–19) from either single-parent or two-parent families who completed both the health examination and health interview surveys in three waves of KNHANES. From this initial group, 250 adolescents were excluded because their parents did not respond to questionnaires regarding depression and Patient Health Questionnaire-9 (PHQ-9). Considering that chronic and severe parental illnesses can affect family dynamics and children’s health [25–29], we excluded 350 participants whose parents had a history of cardiovascular disease, renal failure, cirrhosis, or cancer. The final sample consisted of 4,059 adolescents (Fig. 1).

## Associated factors

### Adolescents’ variables

Participants’ demographic and socioeconomic characteristics — such as age, sex, education level, household income and family type — were obtained from the KNHANES surveys conducted between 2013 and 2021 [26, 30, 31] (Table 1). We selected participants’ weight for their age, smoking habits, alcohol consumption, and physical activity levels as health behavior factors for adolescents’s variables (Table 2). Three factors — subjective health status, stress levels, and depressive mood — were selected as the psychological well-being factors attributable to adolescents (Table 2).

For the health behaviors factors, participants’ weight for their age was classified into three groups (< 5th, 5th -95th, and ≥ 95th percentile) according to the Korean National Growth Chart in 2017 [32]. The KNHANES questionnaires on smoking, alcohol consumption, and physical activity levels were used to categorize the participants’ health behavior statuses. Participants were classified as “ever-smokers” or “never-smokers” based on smoking status (“yes” or “no”) for participants aged 12–18 years, and lifetime smoking status (less than 100 cigarettes, 100 or more cigarettes, or never) for participants aged 19 years or older. Lifetime alcohol consumption was determined by asking whether the participants had ever consumed more than one drink in their lives, with a “yes” or “no” answer. The researchers assessed participants’ annual physical activity levels by categorizing the data into binary variables. This categorization depended on whether the participants met the American College of Sports Medicine (ACSM)’s exercise guidelines, which require at least 150 min per week of moderately-intensive physical activity, more than 75 min per week of vigorously-intensive physical activity, or a balanced combination of the two.

For the psychological well-being factors, the participants’ subjective health statuses were assessed using a single question: “How would you rate your general health?” The answers were divided into two groups: one group contained “very poor” or “poor” answers, with the other group containing “very good,” “good,” or “fair” answers. The adolescents’ stress levels were assessed by asking them about the amount of stress they experienced on a daily basis. Participants who answered “very high stress” or “high stress” were classified into the “high-stress” group, whereas those who answered with “very low stress” or “low stress” were classified into the “low-stress” group. Depressive mood was assessed using a single question: “In the past year, have you experienced a period of two weeks or longer when you felt sad or hopeless almost every day?”, with “yes” or “no” answers.

### Parental depression variables

Information on the diagnosis, treatment, and current depression status of the parents was obtained using medical history questionnaires. Parents with depression were identified based on their response (yes or no) to the question regarding “diagnosis of depression by a doctor.”’ The current depression status of parents was determined by their response (yes or no) to the question about the “current presence of depression.” Additionally, confirmation of parents’ experience with depression treatment was based on their response (yes or no) to the question about “receiving treatment for depression.” To calculate the duration of parental depression, we subtracted the parents’ reported age at the first diagnosis of depression from their age at the time of the questionnaire.

The self-reported depression questionnaire did not include information on the severity of the depressive symptoms. Therefore, we used PHQ-9 scores to determine the severity of depressive symptoms among parents. The internal consistency of the Korean version PHQ-9 was assessed by using the Cronbach’s alpha coefficient, and it was found to be 0.81 [33]. As the PHQ-9 scores were only collected in 2014, 2016, 2018, and 2020, we extracted a subpopulation of 1,714 participants who provided PHQ-9 scores from the final population. We categorized the severity of parental depression as follows: scores of 0–4 presented “none to minimal depression,” scores of 5–9 presented “mild depression,” and scores of 10 or more presented “moderate to severe depression.”

### Data analysis

Descriptive statistical methods were used to provide an overview of the baseline characteristics of the study participants. Categorical variables were analyzed using the chi-squared test, whereas continuous variables were analyzed using the unpaired t-test. We performed univariate logistic regression analysis to assess the association between parental depression and adolescent factors such as heath behaviors factors (abnormal weight for age, smoking, alochol use, physical activity) and psychological factors (subjective health status, stress level, depressive mood). In the multivariate logistic regression analysis, we included fourcovariates — sex of adolescent children, age of adolescent children, household income and family type— to calculate the adjusted odds ratios for adolescent health behaviors and mental health outcomes. Statistical significance was determined by a p-value of less than 0.05, and 95% confidence intervals were reported. All the statistical analyses were performed using SAS version 9.4 (SAS Institute Inc., Cary, NC, USA).

## Results

### Baseline characteristics

The baseline characteristics of the participants are summarized in Table 1. The proportion of adolescents with parental depression was 5.00% (*n* = 203). Among participants with parental depression, there was a higher percentage of households with the lowest income level (< 2,000 — 30.1%) and a higher prevalence of single-parent family structures (26.4%) than those without parental depression (*p* < 0.05).

**Table 1:** Characteristics of adolescents aged from 12 to 19 (*n* = 4059) and their parents. The data is displayed as both a numerical value (N) and a percentage (%), or as the mean plus or minus the standard deviation (mean ± S.D.). ^a^ p-value from chi-square test for categorical variables or unpaired t-test for continuous variables. *P < 0.05

Characteristics	Without parental depression	With parental depression	df	Pearson chi-square value	Cramer’s V or phi value	p ^a^
	3856 (95.0)	203 (5.0)				
Age (years)	15.3 ± 2.3	15.5 ± 2.3	1			0.250
**Sex**						
Male	1999 (51.8)	112 (55.2)	1	0.857	-0.015	0.355
Female	1857 (48.2)	91 (44.8)				
**Weight for age**						
< 5 percentile	2657 (78.5)	137 (78.7)	2	0.029	0.003	0.986
5–95 percentile	483 (14.3)	25 (14.4)				
> 95 percentile	245 (7.2)	12 (6.9)				
**Smoking**						
Non-smoking	2883 (89.6)	142 (88.2)	1	0.316	0.010	0.574
Ever-smoker	335 (10.4)	19 (11.8)				
**Alcohol intake**						
Nondrinker	2623 (73.6)	127 (70.6)	1	0.790	0.015	0.374
Drinker	943 (26.4)	53 (29.4)				
**Education level**						
< Elementary school	1781 (46.2)	89 (43.9)	2	2.493	0.025	0.288
Middle school	1266 (32.8)	62 (30.5)				
> High school	809 (20.0)	52 (25.6)				
**Household monthly income**						
< 2000	381 (9.9)	61 (30.1)	2	81.515	0.142	< 0.0001 *
2000–5000	1870 (48.5)	82 (40.4)				
> 5000	1605 (41.6)	60 (29.5)				
**Family type**						
Two-parent family	3198 (82.9)	142 (73.6)	1	22.308	-0.074	< 0.0001 *
Single-parent family	658 (17.1)	51 (26.4)				
**Parental depression**						
Paternal depression		33 (16.3)				
Maternal depression		178 (87.7)				
Both paternal and maternal depression		8 (3.94)				
**Depression treatment**						
Yes		131 (64.5)				
No		72 (35.5)				
**Current depression**						
Yes		88 (43.3)				
No		115 (56.7)				
**Duration of depression**						
≤ 5 year		92 (45.3)				
> 5 year		111 (54.7)				

### Adolescent health behaviors

In the study results, no significant differences were found in the risks of abnormal weight, smoking, alcohol consumption or physical activity based on parental depression (Table 2). However, boys had higher prevalence rates of smoking, alcohol consumption, and physical activity regardless of parental depression. In addition, no significant differences were observed in the risk of abnormal weight, smoking, alcohol consumption, or physical activity based on the severity of parental depression (Table 3). Although there were no statistically-significant differences in the proportions of abnormal weight, current smokers, alcohol drinkers, and recommended levels of physical activity between the two groups of adolescents with and without parental depression, Table 4 shows that the crude proportions of abnormal weight and alcohol consumption were higher when parents had been treated for depression, when depression was present, and when more than five years had elapsed since the diagnosis of parental depression. Additionally, adolescents with parents who were treated for depression and who were currently depressed had lower levels of physical activity.

### Adolescent mental health

After multivariate adjustments, Table 2 shows that the increased risks of depressive mood were statistically significant in boys with parental depression (aOR = 2.46, 95% Confidential Interval (CI) = 1.22–4.96). The risks of very high and high stress levels for both boys and girls were significantly higher when adolescents had parental depression (stress levels for boys = aOR, 2.20, 95% CI = 1.35–3.58. Stress levels in girls: aOR = 1.66, 95% CI = 1.00–2.75). The risk of very poor or poor subjective health only increased in girls with parental depression (aOR = 2.54, 95% CI = 1.21–5.32).

When parents had moderate to severe depression, the risk of adolescent stress and depressive mood increased significantly after multivariate adjustments (stress: aOR = 1.81, 95% CI = 1.01–3.24; depressive mood: aOR = 2.6, 95% CI = 1.17–5.77 — Table 3). In addition, subjective health and distress were significantly higher among adolescents with parental depression (subjective health: aOR = 1.897, 95% CI = 1.05–3.36; distress: aOR = 1.91, 95% CI = 1.33–2.76 — Table 4). However, depressive mood did not differ significantly between adolescents with and without parental depression (aOR = 1.33, 95% CI = 0.77–2.28).

Among adolescents with parental depression, those with parents who were treated for depression and who currently had depression had significantly higher subjective health statuses and stress levels than.

**Table 2:** Behavioral and mental health factors among adolescents with or without parental depression. The data is displayed as both a numerical value (N), a percentage (%), and odds ratio (95% CI). OR odds ratio; CI confidence interval. ^a^ Abnormal weight for age was determined as being equal to or greater than the 95th percentile or less than the 5th percentile of weight for the age of Korean teenagers. ^b^ Adjusted for age, sex, household monthly income and family type. ^c^ Recommended intensity of physical activity (150 min/week of moderate-intensity physical activity, or 75 min/week of vigorous-intensity physical activity, or the equivalent dose of moderate and vigorous physical activity). *P < 0.05

Category	Sex		Adolescents without parental depression	Adolescents with parental depression
Abnormal weight for age^a^	Boys	N (Proportion, %)	409 (23.0)	20 (20.8)
		OR	1.00	0.75 (0.43 1.32)
		aOR ^b^	1.00	0.71 (0.39 1.27)
	Girls	N (Proportion, %)	319 (19.9)	17 (21.8)
		OR	1.00	1.34 (0.73 2.47)
		aOR ^b^	1.00	1.34 (0.76 2.37)
Ever-smoker, yes	Boys	N (Proportion, %)	242 (14.2)	13 (14.3)
		OR	1.00	1.27 (0.63 2.55)
		aOR ^b^	1.00	1.09 (0.52 2.27)
	Girls	N (Proportion, %)	93 (6.12)	6 (8.57)
		OR	1.00	1.70 (0.69 4.18)
		aOR ^b^	1.00	1.35 (0.54 3.43)
Any alcohol use, yes	Boys	N (Proportion, %)	520 (27.9)	33 (33.0)
		OR	1.00	1.255 (0.79 1.99)
		aOR ^b^	1.00	1.239 (0.69 2.24)
	Girls	N (Proportion, %)	423 (24.9)	20 (25.0)
		OR	1.00	1.21 (0.68 2.13)
		aOR ^b^	1.00	1.02 (0.47 2.23)
Moderate/vigorous intensity physical activity ^c^	Boys	N (Proportion, %)	528 (77.6)	24 (72.7)
		OR	1.00	0.59 (0.26 1.36)
		aOR ^b^	1.00	0.61 (0.27 1.37)
	Girls	N (Proportion, %)	398 (63.0)	22 (64.7)
		OR	1.00	0.97 (0.43 2.17)
		aOR ^b^	1.00	1.04 (0.47 2.27)
Very bad/bad Subjective health status	Boys	N (Proportion, %)	85 (4.55)	5 (5.0)
		OR	1.00	0.97 (0.37 2.55)
		aOR ^b^	1.00	1.03 (0.39 2.72)
	Girls	N (Proportion, %)	84 (4.94)	10 (12.3)
		OR	1.00	3.07 (1.50 6.28)
		aOR ^b^	1.00	2.54 (1.21 5.32) *
Very high/high level stress	Boys	N (Proportion, %)	361 (19.3)	35 (35.0)
		OR	1.00	2.22 (1.37 3.62)
		aOR ^b^	1.00	2.20 (1.35 3.58) *
	Girls	N (Proportion, %)	488 (28.7)	31 (38.8)
		OR	1.00	1.74 (1.05 2.87)
		aOR ^b^	1.00	1.66 (1.00 2.75) *
Depressive mood more than two weeks	Boys	N (Proportion, %)	91 (5.1)	11 (11.6)
		OR	1.00	2.64 (1.29 5.37)
		aOR ^b^	1.00	2.46 (1.22 4.96) *
	Girls	N (Proportion, %)	160 (9.9)	7 (9.2)
		OR	1.00	0.78 (0.33 1.83)
		aOR ^b^	1.00	0.68 (0.28 1.63)

**Table 3:** Behavioral and mental health factors among adolescents according to the severity of the parental depression symptom. The data is displayed as both a numerical value (N), a percentage (%), and odds ratio (95% CI). OR odds ratio; CI confidence interval. ^a^ Depressive symptom severity was based on PHQ-9 score ^b^ Abnormal weight for age was determined as being equal to or greater than the 95th percentile or less than the 5th percentile of weight for the age of Korean teenagers. ^c^ Adjusted for age, sex, household monthly income and family type. ^d^ Recommended intensity of physical activity (150 min/week of moderate-intensity physical activity, or 75 min/week of vigorous-intensity physical activity, or the equivalent dose of moderate and vigorous physical activity). *P < 0.05

	Depressive symptom severity ^a^		
**Characteristic**	**None-to-minimal depression (0–4)** **(n = 1,348)**	**Mild depression (5–9) ** **(n = 270)**	**Moderate-to-severe depression (**≥ **10)** **(n = 96)**
**Abnormal weight for age** ^b^			
N (Proportion, %)	270 (22.9)	56 (23.0)	17 (22.1)
OR	1.00	1.00 (0.68 1.46)	1.00 (0.55 1.79)
aOR ^c^	1.00	0.96 (0.64 1.43)	0.97 (0.52 1.80)
**Ever-smoker, yes**			
N (Proportion, %)	107 (9.5)	26 (11.5)	4 (5.3)
OR	1.00	1.47 (0.88 2.46)	0.65 (0.19 2.24)
aOR ^c^	1.00	1.43 (0.82 2.48)	0.55 (0.15 1.98)
**Any alcohol use, yes**			
N (Proportion, %)	331 (26.4)	78 (31.2)	22 (26.2)
OR	1.00	1.19 (0.84 1.68)	1.04 (0.58 1.85)
aOR ^c^	1.00	1.40 (0.90 2.17)	0.98 (0.41 2.36)
**Moderate/vigorous intensity physical activity**. ^d^			
N (Proportion, %)	493 (71.4)	94 (68.6)	26 (66.7)
OR	1.00	0.93 (0.59 1.45)	0.68 (0.31 1.51)
aOR ^c^	1.00	0.93 (0.59 1.48)	0.80 (0.39 1.64)
**Very bad/bad Subjective health status**			
N (Proportion, %)	63 (5.0)	13 (5.2)	8 (9.4)
OR	1.00	0.82 (0.43 1.55)	2.07 (0.83 5.18)
aOR ^c^	1.00	0.85 (0.50 1.59)	2.02 (0.81 5.00)
**Very high/high level stress**			
N (Proportion, %)	302 (24.1)	64 (25.6)	27 (32.1)
OR	1.00	1.10 (0.77 1.59)	1.89 (1.08 3.29)
aOR ^c^	1.00	1.10 (0.77 1.58)	1.81 (1.01 3.24) *
**Depressive mood more than two weeks**			
N (Proportion, %)	74 (6.6)	23 (10.1)	11 (14.7)
OR	1.00	1.64 (0.95 2.83)	2.87 (1.35 6.09)
aOR ^c^	1.00	1.62 (0.93 2.85)	2.60 (1.17 5.77) *

**Table 4:** Behavioral and mental health factors among adolescents dependent on depression diagnosis, depression treatment, current depression and the time since depression diagnosis. The data is displayed as both a numerical value (N), a percentage (%), and odds ratio (95% CI). OR odds ratio; CI confidence interval. ^a^ Abnormal weight for age was determined as being equal to or greater than the 95th percentile or less than the 5th percentile of weight for the age of Korean teenagers. ^b^ Adjusted for age, sex, household monthly income and family type. ^c^ Recommended intensity of physical activity (150 min/week of moderate-intensity physical activity, or 75 min/week of vigorous-intensity physical activity, or the equivalent dose of moderate and vigorous physical activity). *P < 0.05

Characteristics	Adolescents without parental depression (*n* = 3856)	Adolescents with parental depression (*n* = 203)	Adolescents with parental depression					
			Depression treatment		Current depression		Duration of depression	
			No **(n = 131)**	Yes **(n = 72)**	No **(n = 88)**	yes **(n = 115)**	≤ 5 year **(n = 92)**	> 5 year **(n = 111 )**
**Abnormal weight for age** ^a^								
N (Proportion, %)	728 (21.5)	37 (21.3)	18 (15.9)	19 (31.1)	13 (17.8)	24 (23.8)	15 (18.8)	22 (23.4)
OR	1.00	0.98 (0.63 1.54)	0.76 (0.41 1.40)	1.44 (0.74 2.79)	0.94 (0.47 1.89)	1.02 (0.57 1.81)	0.78 (0.42 1.45)	1.17 (0.64 2.15)
aOR ^b^	1.00	0.94 (0.56 1.48)	0.73 (0.40 1.33)	1.35 (0.66 2.75)	0.93 (0.48 1.82)	0.94 (0.51 1.72)	0.77 (0.41 1.45)	1.09 (0.59 2.00)
**Ever-smoker, yes**								
N (Proportion, %)	335 (10.4)	19 (11.8)	11 (10.5)	8 (14.3)	7 (10.6)	12 (12.6)	10 (13.3)	9 (10.5)
OR	1.00	1.43 (0.81 2.51)	0.94 (0.46 1.94)	2.37 (1.05 5.37)	0.88 (0.35 2.21)	1.85 (0.94 3.65)	2.19 (1.05 4.58)	0.83 (0.37 1.86)
aOR ^b^	1.00	1.19 (0.65 2.16)	0.83 (0.38 1.83)	1.83 (0.78 4.27)	0.90 (0.31 2.63)	1.36 (0.68 2.74)	1.95 (0.91 4.16)	0.67 (0.27 1.65)
**Any alcohol use, yes**								
N (Proportion, %)	943 (26.4)	53 (29.4)	34 (29.1)	19 (30.2)	22 (28.6)	31 (30.1)	21 (25.6)	32 (32.6)
OR	1.00	1.24 (0.88 1.75)	1.17 (0.78 1.76)	1.36 (0.74 2.51)	1.15 (0.67 1.95)	1.31 (0.84 2.05)	1.19 (0.70 2.04)	1.28 (0.83 1.98)
aOR ^b^	1.00	1.16 (0.73 1.85)	1.21 (0.67 2.18)	1.10 (0.52 2.33)	1.13 (0.50 2.52)	1.19 (0.69 2.07)	1.21 (0.63 2.33)	1.12 (0.59 2.16)
**Moderate/vigorous intensity physical activity** ^c^								
N (Proportion, %)	926 (70.6)	46 (68.7)	29 (74.4)	17 (60.7)	20 (74.1)	26 (65.0)	19 (65.5)	27 (71.1)
OR	1.00	0.76 (0.43 1.35)	1.36 (0.62 3.00)	0.44 (0.20 0.96)	1.39 (0.54 3.59)	0.55 (0.28 1.09)	0.69 (0.34 1.38)	0.82 (0.35 1.90)
aOR ^b^	1.00	0.84 (0.47 1.48)	1.50 (0.67 3.39)	0.48 (0.22 1.03)	1.52 (0.57 4.08)	0.61 (0.31 1.19)	0.74 (0.36 1.55)	0.92 (0.41 2.07)
**Very bad/ bad Subjective health status**								
N (Proportion, %)	169 (4.74)	15 (8.29)	7 (5.98)	8 (12.5)	5 (6.49)	10 (9.62)	6 (7.23)	9 (9.18)
OR	1.00	1.94 (1.08 3.47)	1.33 (0.59 3.03)	2.95 (1.35 6.44)	1.38 (0.52 3.65)	2.36 (1.18 4.75)	1.87 (0.77 4.56)	2.00 (0.96 4.16)
aOR ^b^	1.00	1.88 (1.05 3.36) *	1.26 (0.55 2.89)	3.01 (1.39 6.54) *	1.29 (0.47 3.55)	2.36 (1.19 4.67) *	1.80 (0.72 4.46)	1.95 (0.94 4.06)
**Very high/high level stress**								
N (Proportion, %)	849 (23.8)	66 (36.7)	44 (37.6)	22 (34.9)	26 (33.8)	40 (38.8)	25 (30.5)	41 (41.8)
OR	1	1.93 (1.36 2.76)	2.02 (1.28 3.19)	1.79 (1.06 3.05)	1.75 (1.01 3.02)	2.08 (1.33 3.24)	1.17 (0.68 2.01)	2.85 (1.81 4.48)
aOR ^b^	1	1.91 (1.33 2.76) *	2.01 (1.25 3.22) *	1.76 (1.03 3.02) *	1.72 (0.98 3.01)	2.08 (1.31 3.28) *	1.12 (0.64 1.96)	2.90 (1.83 4.57) *
**Depressive mood more than two weeks**								
N (Proportion, %)	251 (7.35)	18 (10.5)	11 (9.65)	7 (12.3)	5 (6.76)	13 (13.4)	9 (11.4)	9 (9.78)
OR	1.00	1.46 (0.86 2.49)	1.42 (0.71 2.82)	1.55 (0.69 3.46)	0.81 (0.31 2.12)	2.01 (1.07 3.78)	1.31 (0.59 2.91)	1.60 (0.80 3.19)
aOR ^b^	1.00	1.33 (0.77 2.28)	1.31 (0.64 2.65)	1.36 (0.61 3.05)	0.74 (0.28 1.94)	1.84 (0.96 3.50)	1.13 (0.50 2.55)	1.51 (0.76 3.02)

those without parental depression (Table 4). The risk of very high or high stress levels was also significantly higher when parents were not treated for depression (aOR = 2.01, 95% CI = 1.25–3.22). If the duration of depression of parents exceeds 5 years, stress levels were significantly high (aOR = 2.29, 95% CI = 1.83–4.57).

## Discussion

This study shows that parental depression can have the association of several aspects of adolescents’ mental health, including depressive mood, stress levels, and subjective health status. These effects were not only influenced by the adolescents’ gender, but also by factors such as parents’ current depression, treatment for depression, severity of depression, and time since diagnosis.

In the present study, boys whose parents were diagnosed with depression showed an higher risk of experiencing depressive mood and elevated stress than in girls. These findings differ from previous studies suggesting that girls are more vulnerable to parental depression [3, 34, 35], possibly due to their heightened sensitivity to interpersonal relationships and susceptibility to their parents’ emotional state [36–38]. Specifically, in Asian cultures, there is an expectation for children to financially support their caregivers, with male children, in particular, having significant responsibility for economic support [39]. Therefore, male children may face challenges in discussing their difficulties with caregivers due to these responsibility for supporting financial burdens. While previous research indicated that male children of parents with illness exhibit more externalizing problem behaviors compared to female children [40, 41], this study did not observe problematic behaviors, which is considered a limitation of the research. Consistent with reports indicating a higher suicide rate among Korean boys compared to girls [42], these findings underscore the need for careful management of boys with parental depression to address their mental health conditions.

Aligned with findings indicating that chronic and severe parental depression symptoms have more adverse effects on adolescents’ mental health [10, 43–45], this study confirms that severe parental depression symptoms, the duration of depression longer than 5 years, and current depression are associated with an increased risk of adverse adolescent mental health. These results in a Korean population-based study can be explained by the biopsychosocial model of depression [46, 47], referred to as thea association of parental depression on children’s mental health being a complex interaction of factors such as biological vulnerability, psychological factors, and social factors. First, as a biological vulnerability factor, children of parents with depression have a higher genetic vulnerability to depression [48]. For instance, Weissman et al. reported that, after following children for 20 years, those with depressed parents had more than three times the risk of developing depression and anxiety disorders compared to children without parental depression [6]. Second, as a psychological factor, severe and prolonged depressive symptoms in parents may interfere with their ability to fulfill their parenting role, leading to withdrawal or intrusiveness, which may exacerbate depression and stress in adolescents [49–51]. In addition, parental depression symptoms negatively affect parenting styles, worsen parent-child relationship interactions, and increase stress in children [52–54]. Third, as a social factor, parents with depression are more likely to experience unemployment and have lower household incomes [55]. Consequently, the deterioration of the economic situation for parents with depression can make it difficult for their children to find employment themselves or access educational opportunities, which may negatively impact their mental health and academic performance [56]. Especially, in light of the competitive social atmosphere prevalent in Asian cultures, the pressure of meeting parental expectations and parental interference in academic matters could serve as significant stressors for adolescents in Asian countries [57]. Therefore, our findings highlight the importance of comprehensive approach and earlier interventions in preventing parental depression from becoming more chronic and severe symptoms.

Contrary to previous research suggesting that treating depression in parents has a beneficial effect on the mental well-being of adolescents [8, 9], this study found that the stress levels and subjective health status of adolescents whose parents received treatment for depression were more vulnerable. Table s1 shows that the proportion of patients with mild and/or moderate to severe depression was higher in the treatment group (see Additional file 1). Despite South Korea being among the OECD countries with the highest annual outpatient visits and inpatient admissions, psychiatric clinic visits are relatively low due to the social stigma associated with mental illness [58]. Consequently, delayed treatment by parents with depression may lead to an increased severity of depressive symptoms, which may negatively affect their adolescent children. Therefore, efforts are required to improve negative perceptions about mental illness to ensure that parents with mental health conditions receive timely psychiatric treatment.

Although parental depression did not have a significant effect on adolescents’ negative health behaviors and physical activity, the results showed that adolescents with parental depression had a poorer subjective health status. While subjective health status serves as a factor reflecting how adolescents comprehensively perceive their health conditions and is linked to both mental and physical health [59, 60], it may not accurately represent the individual states of participants concerning variations in depressive symptoms and stress levels. However, consistent with parental depression potentially leading to adolescents’ anxiety or concerns about their own physical health [61], these findings suggest the possibility of hypochondriasis [62], considering that physical symptoms (loss of appetite, digestive problems, headaches, etc.) can manifest in individuals with depressive mood and psychological distress. Therefore, interventions that can help ensure the health status of adolescents should be considered along with the treatment of parental depression, as it may exacerbate adolescents’ concerns or anxiety about their subjective health status.

Our study has several strengths, including the use of nationwide data sources, which facilitates comparisons of outcomes within a wider adolescent population. In addition, the use of a community-based design enhanced the ability of the study to reflect the real-life circumstances of families dealing with depression, unlike a hospital-based study. Furthermore, we had comprehensive access to information regarding the physical and psychosocial welfare of adolescents with and without parental depression. The results of our study have significant implications for future interventions, emphasizing the importance of prioritizing the screening and management of adolescents’ mental health after diagnosing parental depression while also crucially evaluating their health behaviors. Future research should focus on exploring interventions that address the needs of families with parents with depression to reduce the psychological risks for their adolescent children.

## Limitations

Our study has several limitations that need to be considered. First, the assessment of adolescents’ mental health status was limited to a single question, which posed challenges in accurately assessing and categorizing specific psychiatric diagnoses, such as anxiety disorder, bipolar disorder, and major depressive disorder. Second, although the clinical manifestations and hospitalization of parental depression may allow researchers to better confirm the difference between adolescents with parental depression and the general population, we did not consider the duration and frequency of parental symptoms of depression, hospitalization rates for depression, and treatment options for depression, which were not captured by the questionnaires. Finally, we encountered difficulties in obtaining relevant information about adolescents’ medical histories, including psychiatric conditions, other chronic illnesses, school absenteeism, and academic performance.

## Conclusions

We concluded that Korean adolescents with parental depression have poorer mental health status than whose parents did not have mental health issues. Specifically, we identified that depressive mood over two weeks among adolescents with parental depression was more prevalent in boys and in cases where parents exhibited moderate-to-severe depressive symptoms. Additionally, when parental depression was present and the time since depression diagnosis was more than five years, adolescents with parental depression exhibited even poorer subjective health status and higher stress levels. However, adolescents with parental depression did not differ in their health behaviors compared to their general peers. These results highlight the need for tailored, community-based social support for adolescents with parental depression to promote their psychological well-being and encourage healthy behaviors as they transition into adulthood.

### Electronic supplementary material

Below is the link to the electronic supplementary material.

Supplementary Material 1

## Data Availability

The KNHANES database which support this study are available from the KNHANES website (knhanes.kdca.go.kr, Korea Disease Control and Prevention Agency).

## Abbreviations

KNHANES: Korean National Health and Nutrition Examination Survey
CI: Confidence Interval
OR: Odd Ratio
aOR: adjusted Odd Ratio
WHO: World Health Organization
OECD: Organization for Economic Co-operation and Development
ACSM: American College of Sports Medicine
