# Supplementary material for: Association of parental depression with adolescent children’s psychological well-being and health behaviors
Source: BMC Public Health. 2024 May 27;24:1412. doi: 10.1186/s12889-024-18337-9 (PMC11129386; doi:10.1186/s12889-024-18337-9)
Supplement: Supplementary file 1 — Supplementary Material 1 [file 12889_2024_18337_MOESM1_ESM.docx]

[**Appendix] Table 1.** Number and proportion of the depression severity on adolescents with/without parental depression, depression treatment, current depression and time since diagnosis year. The data is displayed as both a numerical value (N), a percentage (%), and odds ratio (95% CI). OR odds ratio; CI confidence interval. . ^a^ Depressive symptom severity was based on PHQ-9 score

| **Depressive symptom severity** ^a^ | **Adolescent without Parental depression** | **Adolescent with Parental Depression** | **Adolescent with Parental Depression** | | | | | |
| --- | --- | --- | --- | --- | --- | --- | --- | --- |
|  |  |  | **Depression Treatment** | | **Current depression** | | **Duration of depression** | |
|  |  |  | **No** | **Yes** | **No** | **Yes** | ≤ **5 year** | **> 5 year** |
| **None-to-minimal depression (0**–**4)** | 1317 (80.9) | 31 (36.1) | 23 (47.9) | 8 (21.1) | 21 (75.0) | 10 (17.2) | 14 (35.9) | 17 (36.2) |
| **Mild depression (5**–**9)** | 244 (15.0) | 26 (30.2) | 12 (25.0) | 14 (36.8) | 7 (25.0) | 19 (32.8) | 12 (30.8) | 14 (29.8) |
| **Moderate-to-severe depression (**≥**10)** | 67 (4.1) | 29 (33.7) | 13 (27.1) | 16 (42.1) | n/a | 29 (50.0) | 13 (33.3) | 16 (34.0) |
